# Functional Characterization and Heterogeneity Analysis of Ribosomal Proteins in Mouse Preimplantation Embryos

**DOI:** 10.1096/fj.202500574RR

**Published:** 2025-05-27

**Authors:** Seung Jae Lee, Dayeon Kim, Gwidong Han, Seung Pyo Hong, Inchul Choi, Chunghee Cho

**Affiliations:** ^1^ Department of Life Sciences Gwangju Institute of Science and Technology Gwangju Republic of Korea; ^2^ Department of Animal and Dairy Sciences Chungnam National University Daejeon Republic of Korea

**Keywords:** *de novo* protein synthesis, morula to blastocyst transition, mouse preimplantation embryo, protein folding, ribosomal protein, ribosome heterogeneity

## Abstract

Translational control is important during the mammalian preimplantation phase, when maternal RNAs and proteins are degraded and *de novo* synthesis of RNAs and proteins increases. Proteins are synthesized in ribosomes, which are assembled from ~82 ribosomal proteins (RPs). The function of ribosomes varies depending on the resident RPs, suggesting that ribosome heterogeneity can lead to functional specialization. Only a few studies have investigated the function of RPs during preimplantation embryonic development. Here, we performed functional analyses on six RP‐encoding genes—*Rpl4*, *Rps9*, *Rps11*, *Rpl13a*, *Rpl19*, and *Rpl39*—in mouse preimplantation embryos. Knockdown (KD) of each of these RP genes, except *Rpl39*, affected morula‐to‐blastocyst transition, producing phenotypes that varied somewhat in their details. *Rpl4‐*, *Rpl13a‐*, and *Rpl19*‐KD embryos showed fragmentation and strong arrest of cell proliferation, whereas *Rps9‐* and *Rps11*‐KD embryos showed severe fragmentation with relatively weak arrest of cell proliferation. In the case of *Rpl39*, single‐KD embryos developed normally, but double‐KD embryos with its paralog *Rpl39*‐like (*Rpl39l*) inhibited normal blastocyst development. Protein misfolding signals were also activated in *Rpl39*‐KD and *Rpl39l* + *Rpl39* double‐KD embryos, confirming a previous finding that RPL39 and RPL39L are associated with ribosome exit tunnels. Our results suggest the presence of different groups of proteins that require an RPL39‐containing ribosome or RPL39/RPL39L‐containing ribosome for correct folding in early embryos. Taken together, the results of the present study demonstrate that ribosomal proteins are fundamentally important for normal blastocyst formation and development, but not all ribosomal proteins contribute equally to embryonic development, providing a novel example of ribosome heterogeneity in preimplantation embryos.

## Graphical abstract

We selected and investigated six ribosomal proteins in the early mouse embryo. Knocking down these ribosomal proteins generally had inhibitory effects on the morula‐to‐blastocyst transition, but the details and degree of these effects varied. This provides a new example of ribosome heterogeneity in preimplantation embryos.

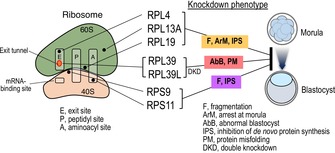

## 1 Introduction

Early mammalian embryonic development, constituting the period from fertilization to implantation, is the process by which the embryo develops from a zygote to blastocyst. During this period, a sequence of molecular and cellular events occurs. First, the zygote divides by cleavage without cell growth [1], a series of divisions that give rise to 8‐ to 16‐cell embryos. At the eight‐cell stage, two major morphological changes occur: compaction and polarization. Compaction causes each blastomere to become tightly and maximally attached, making them morphologically indistinguishable. This compacted mass of blastomeres is called a morula. Simultaneously, cell polarization begins, causing uneven distribution of certain cellular components along the apical‐basal axis of the cells. Subsequently, cavitation creates a fluid‐filled space within the embryo, leading to the formation of the blastocyst for implantation. Apically polarized outer cells differentiate into trophectoderm (TE), whereas inner cells lacking apical polarity differentiate into the inner cell mass [2, 3, 4, 5, 6]. These developmental processes are tightly controlled through regulation of gene expression. The embryo develops using RNAs and proteins stored in the oocyte until the two‐cell stage after fertilization in mice. By the late two‐cell stage, most maternal RNAs and proteins have been degraded, and the amount of newly synthesized RNAs and proteins increases through zygotic genome activation. These new transcripts and proteins play crucial roles in the development of the morula and blastocyst [7, 8]. Several recent studies have shown that deficiencies in genes known to regulate protein translation, including ribosomal proteins (RPs), cause defects in early embryonic development [9, 10, 11, 12].

Proteins are translated by a giant molecular machine called a ribosome. The ribosome consists of two subunits: the small subunit (SSU) and the large subunit (LSU). Each subunit consists of ribosomal RNAs (rRNAs) and RPs; in eukaryotes, there are 33 RPs for the SSU (RPSs) and 49 RPs for the LSU (RPLs) [13]. These RPs are known to be important for ribosome assembly [14]. Although ribosomes in general are considered to share the capacity to regulate the translation of all proteins, recent studies have found that some ribosomes are heterogeneous and selectively translate different proteins [15, 16]. This ribosome heterogeneity is attributable to the RP components of the ribosome, and has been characterized as reflecting RP stoichiometry, RP composition, and the existence of RP paralogs [17]. For instance, RPL38‐deficient ribosomes can fully translate global genes but are unable to translate homeobox genes [18]. In mouse embryonic stem cells (mESCs), distinct subpools of mRNAs are translated differently depending on whether the ribosome contains RPL10A or RPS25 [19]. In addition to this ribosome heterogeneity, recent studies have shown that RPs have extra‐ribosomal functions. Loss of RPS6, RPS14, or RPS19 causes cells to arrest in the G0‐G1 phase and reduces the representation of S & G2‐M phase cells [20, 21]. RPS3A enhances the activity of nuclear factor kappa‐light‐chain‐enhancer of activated B cells (NF‐κB), whereas RPS19 inactivates extracellular signal‐regulated kinases (ERK) and NF‐κB signaling [22, 23]. RPL5, RPL11, RPL23, and RPS7 suppress tumorigenesis, whereas RPS13 accelerates tumorigenesis [24, 25]. Therefore, although RPs are clearly essential for ribosome assembly and function, their extra‐ribosomal functions should not be overlooked. Despite their importance, only a few studies have investigated RPs in preimplantation embryonic development, where protein synthesis is critical [9, 26].

In this study, we randomly selected several RPs that could reasonably (though not necessarily) be considered representative of all RPs in the early mouse embryo and investigated their characteristics and functions. Knocking down these RPs generally had inhibitory effects on the morula‐to‐blastocyst transition, but the details and degree of these effects varied. In particular, we found that the functions of RPL39 and its paralog, RPL39L, differed from those of other RPs. Our study newly discovered RPs involved in ribosome heterogeneity and suggests that the functional diversity of RPs may be somewhat prevalent in mouse preimplantation embryos.

## 2 Materials and Methodologies

### 2.1 Collection and Culture of Mouse Embryos

Oocytes and embryos were collected from superovulated mice following previously published methods [27]. Briefly, B6D2F1 females were superovulated at 8–10 weeks of age by intraperitoneal injection of 7.5 IU pregnant mare serum gonadotropin (PMSG; Prospec, Rehovot, Israel), followed 48 h later by injection of 7.5 IU human chorionic gonadotropin (hCG; Prospec). Superovulated females were mated with B6D2F1 males, and fertilized embryos were collected from the oviduct by dissecting ampullae at 18 h post‐hCG injection. Cumulus cells were removed by pipetting in hKSOM (KSOM with HEPES) containing hyaluronidase (0.3 mg/mL). Thereafter, embryos were cultured in potassium‐supplemented simplex optimized medium (KSOM) at 37°C, covered by mineral oil (Vitrolife, Gothenburg, Sweden), in a humidified atmosphere of 5% CO2 and 5% O_2_ balanced with N_2_. Germinal vesicle (GV) oocytes were collected at 48 h post‐PMSG injection, and metaphase II (MII) oocytes were collected at 20 h post‐hCG injection without mating. One‐cell, 2‐cell, 4‐cell, 8‐cell, morula, and blastocyst embryos were collected at 20, 48, 64, 70, 90, and 120 h post‐hCG injection, respectively. All animal experiments were performed according to the guidelines of the Institutional Animal Care and Use Committee of Gwangju Institute of Science and Technology (GIST; Permit number: GIST‐2023‐004).

### 2.2 Generation of Double‐Stranded RNA

Double‐stranded RNA (dsRNA) regions were generated using a pBlueScript II KS vector following previously published methods [27]. dsRNA regions in enhanced green fluorescent protein (EGFP) and each RP were generated by polymerase chain reaction (PCR) using mouse embryo cDNA. Sense and antisense templates of synthesized dsRNA regions were cloned into a pBlueScript II KS vector. Each strand sequence, including the T7 promoter, was amplified, and each amplified strand was purified using a LaboPass PCR Purification Kit (Cosmogenetech, Seoul, Republic of Korea) and synthesized into RNA using the HiScribe T7 High Yield RNA Synthesis Kit (New England Biolabs [NEB], Ipswich, MA, USA). Thereafter, DNA templates were removed by DNase I (NEB) treatment. For annealing, equimolar quantities of sense and antisense RNA were mixed in annealing buffer (20 mM Tris, pH 7.4, 0.2 mM EDTA) at 80°C for 10 min, and incubated for 3 h in hot distilled water that was allowed to gradually cool at room temperature. Contaminating single‐stranded RNAs and proteins in dsRNA samples were then removed by treating preparations with 1 μg/mL RNase A (MilliporeSigma, Burlington, MA, USA) and 2 μg/mL proteinase K (Geneall, Seoul, Republic of Korea), respectively, for 30 min at 37°C. Each dsRNA was subsequently extracted with LiCl and precipitated with ethanol.

### 2.3 Microinjection

Injection micropipettes were generated from borosilicate capillary tubes using a Sutter p‐97 glass puller (Sutter Instruments, Novato, CA, USA). The injection holder was handcrafted from a borosilicate capillary tube. Approximately ~5 pL of each dsRNA was injected into the cytoplasm of a zygote (20 h post‐hCG injection) using an IM300 microinjector (Narishige, Tokyo, Japan) mounted on an inverted laboratory microscope (Leica Microsystems, Wetzlar, Germany). Microinjections were performed in hKSOM.

### 2.4 RNA Extraction and Reverse Transcription PCR (RT‐PCR)

Total RNA was extracted from brain, heart, lung, liver, spleen, kidney, muscle, ovary (female), and testis (male) of mice using TRIzol (Molecular Research Center, Cincinnati, OH, USA) according to the manufacturer's protocol. cDNA was synthesized from total RNA of each tissue using Omniscript reverse transcriptase (Qiagen, Germantown, MD, USA) with random hexamers and oligo dT (Promega). mRNA was isolated from 15 to 30 embryos using a Dynabead RNA Isolation Kit (Thermo Fisher Scientific, Waltham, MA, USA) according to the manufacturer's instructions and synthesized into cDNA using a Sensiscript RT kit (Qiagen, Germantown, MD, USA) with random hexamer and oligo dT primers. RT‐PCR was conducted using the primers listed in Table S1, with glyceraldehyde‐3‐phosphate dehydrogenase (*Gapdh*) used as a loading control.

### 2.5 Real‐Time Quantitative RT‐PCR (qRT‐PCR)

mRNA was isolated from 15 to 30 embryos using a Dynabead RNA Isolation Kit and synthesized into cDNA as described above. qRT‐PCR was conducted using 20 μL of Sybr Green Taq polymerase mix (Enzynomics, Daejeon, Republic of Korea), 150–200 ng of cDNA, and the primers listed in Table S1. Relative gene expression levels were calculated using the 2^−ΔΔCt^ method and normalized to *Gapdh* levels. Values are presented as means ± SEM. Two‐tailed Student's *t* tests were used for statistical analysis, and a *p* < 0.05 was considered a significant difference.

### 2.6 O‐Propargyl‐Puromycin Assay

O‐propargyl‐puromycin (OPP) was added to embryos in KSOM to a final concentration of 20 μM. Embryos in the negative control group were co‐treated with cycloheximide, added to a final concentration of 50 μg/mL. After incubation for 30 min at 37°C, embryos were washed with 0.1% bovine serum albumin (BSA) and fixed by incubating with 3.7% formaldehyde (MilliporeSigma, Burlington, MA, USA) for 20 min at room temperature. Fixed embryos were washed in 0.1% BSA and permeabilized with 0.25% Triton X‐100 for 30 min at room temperature. After washing again, embryos were incubated in OPP reaction cocktail (prepared according to the manufacturer's instructions) for 30 min at room temperature. After OPP detection, embryos were washed with a rinse buffer and stained with Hoechst 33342 (MilliporeSigma) for 25 min at room temperature. Stained embryos were imaged with an Olympus FV3000RS confocal laser‐scanning microscope. Fluorescence intensity was quantified using Image J.

### 2.7 5‐Ethynyl‐2‐Deoxyuridine Assay

After preincubating 5‐ethynyl‐2‐deoxyuridine (EdU; 10 μM) in KSOM at 37°C for 2 h, embryos were cultured in KSOM with EdU for 4 h and then fixed by incubating in 3.7% formaldehyde (MilliporeSigma, Burlington, MA, USA) in PBS containing 0.1% BSA for 30 min at room temperature. Embryos were permeabilized using 0.25% Triton X‐100 in PBS with 0.1% BSA for 30 min at room temperature, then blocked by incubating in 5% BSA overnight at 4°C. EdU incorporated into *de novo*‐synthesized DNA was detected using a Click‐iT EdU Alexa Fluor 488 Imaging Kit (Thermo Fisher Scientific, Waltham, MA, USA). Embryos were incubated in Click‐iT reaction cocktails containing 0.2% BSA for 30 min at room temperature. For counting nuclei, embryos were stained with the nuclear dye, Hoechst 33342 (MilliporeSigma, Burlington, MA, USA), in 3% BSA with 0.1% Triton X‐100 for 30 min at room temperature. The percentage of EdU‐positive blastomeres in each embryo was counted in Z‐projection images, and fluorescence intensity was quantified using Image J.

### 2.8 TUNEL Assay

TUNEL assays were performed using an in situ cell death detection kit (MilliporeSigma, Burlington, MA, USA). Embryos were fixed by incubating in 3.7% formaldehyde (MilliporeSigma, Burlington, MA, USA) in PBS containing 0.1% BSA for 30 min at room temperature and were then permeabilized by incubating in PBS containing 0.25% Triton X‐100 and 0.1% BSA for 30 min at room temperature. Embryos were blocked in 5% BSA overnight at 4°C. As a positive control, some embryos were incubated in 100 U/mL DNase I with 0.1% BSA for 1 h at 37°C. Thereafter, embryos were incubated in TUNEL reaction mixture (Roche, Basel, Switzerland) containing 0.1% BSA at 37°C for 60 min. For counting nuclei, embryos were stained with Hoechst 33342 (MilliporeSigma, Burlington, MA, USA) in 3% BSA with 0.1% Triton X‐100 for 30 min at room temperature. The total percentage of TUNEL‐positive nuclei in each embryo was counted in Z‐projection images.

### 2.9 Statistical Analysis

All experiments were performed at least in triplicate. Data are presented as means ± SD unless otherwise indicated. Two‐tailed Student's *t* tests were used for statistical analyses, and a *p* < 0.05 was considered significantly different. All numerical values are presented in Table S2.

## 3 Results

### 3.1 Selection and Expression of RP Genes

**FIGURE 1:**
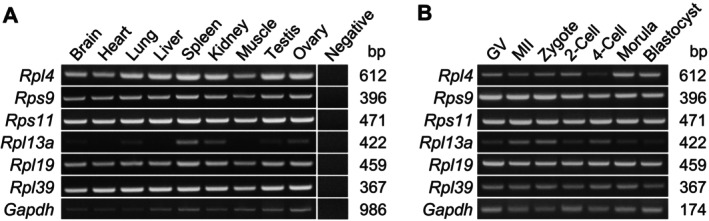
Expression pattern of RP genes in adult mouse tissues and mouse preimplantation embryos. RT‐PCR was performed using cDNA of (A) various tissues and (B) preimplantation embryos, including oocytes. *Gapdh* was used as a loading control. GV, germinal vesicle oocyte; MII, metaphase II egg; Negative, no template.

RNA and protein levels in preimplantation embryos change significantly during the transition from morula to blastocyst, with an increase in the amount of RPs in particular. However, not all RPs exhibit the same level of expression in the embryos (Figure S1); thus, we hypothesized that the function and importance of some RPs would differ in the early mouse embryo. In an attempt to comprehensively study the functions of RPs in mouse preimplantation embryos, we selected six RPs—*Rpl4*, *Rps9*, *Rps11*, *Rpl13a*, *Rpl19*, and *Rpl39*—out of a total of approximately 82 known RPs, taking into account the following criteria: expression levels in mouse preimplantation embryos based on transcriptome and proteome data [8, 28], locations within the ribosome structure [29], and relative levels in monosomes and polysomes [30]. These six genes were chosen randomly but were considered to be generally representative of all RPs in that they are not clustered around any particular feature in the above list of criteria (Figure S1). To investigate the expression pattern of each RP gene, we performed RT‐PCR in various adult mouse tissues. All selected RP genes, with the exception of *Rpl13a*, which was expressed in only a few tissues (including the ovary), were widely expressed in adult mouse tissues (Figure 1A). All six RP genes were expressed ubiquitously in oocytes and preimplantation embryos (Figure 1B), suggesting that their activity and function are involved in early embryonic development.

### 3.2 Development of RP Gene Knockdown Embryos

**FIGURE 2:**
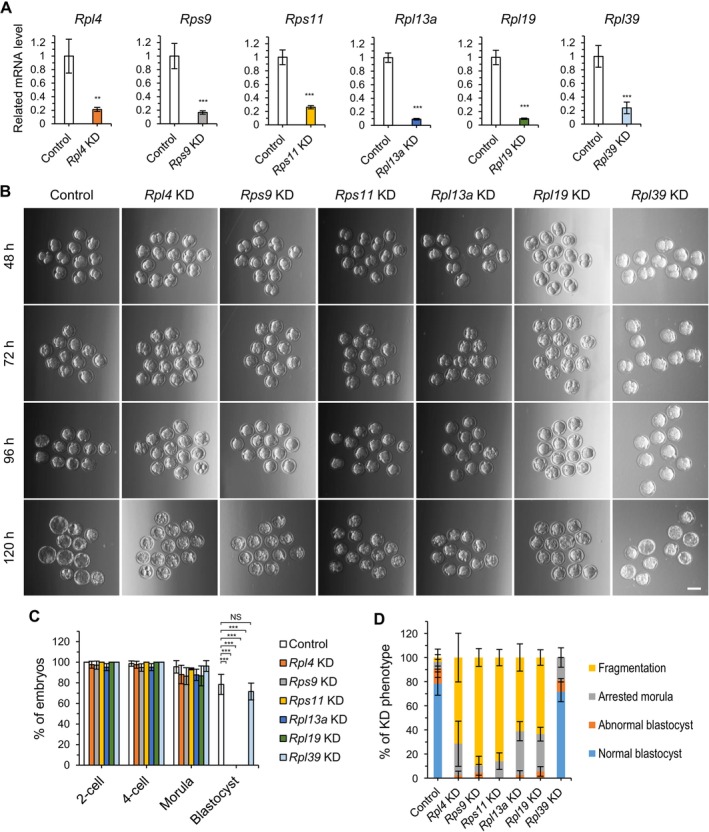
Knockdown analysis of embryos microinjected with dsRNA for each of six RPs. Zygotes (20 h post‐hCG injection) were injected with 1 mg/mL of RP gene dsRNA or 1 mg/mL of EGFP dsRNA (injection control). (A) Expression levels of RP genes, normalized to that of *Gapdh*. cDNA was synthesized from morula stage embryos at 90 h post‐hCG injection. Values represent means ± SEM; ***p < 0.001, **p < 0.01 (experiments were repeated three times, Student's *t* test). (B) Micrographs showing each stage in the development of control and six RP gene‐knockdown KD embryos at 48, 72, 96, and 120 h post‐hCG injection. Scale bar, 100 μm. (C) Embryo development monitored from the two‐cell to the blastocyst stage (up to 120 h post‐hCG injection). EGFP dsRNA‐injected embryos (*n* = 193) and KD embryos for *Rpl4* (*n* = 42), *Rps9* (*n* = 38), *Rps11* (*n* = 48), *Rpl13a* (*n* = 39), *Rpl19* (*n* = 36), and *Rpl39* (*n* = 32) were used for the analysis. Data are presented as means ± SEM; ***p < 0.001 (three biological replicates, Student's *t* test). (D) Control and RP gene‐KD embryos separated based on morphology at the blastocyst stage. Error bars represent SEM.

To investigate the importance of the six RPs in early embryonic development, we performed qRT‐PCR analysis in knockdown (KD) embryos, created by microinjecting dsRNA into mouse zygotes. This analysis showed that the expression of all six RPs was reduced by more than 77% and 74% at the two‐cell and morula stages, respectively, compared with control embryos injected with EGFP dsRNA (Figures 2A and S2). This suggests that the KD effect of dsRNA persists until the late preimplantation stage. Next, we cultured embryos injected with EGFP dsRNA (control) or dsRNAs of each gene (KD) for the same time as required for normal embryos to reach the blastocyst stage and monitored their development. All RP gene‐KD embryos and controls developed to two‐cell, four‐cell, and morula stages at similar rates. However, all RP gene‐KD embryos except *Rpl39*‐KD failed to develop into blastocysts (Figure 2B,C and Table 1). This abnormality in morula‐to‐blastocyst transition may reflect a surge in RP expression during the morula stage, as revealed by transcriptomic and proteomic data [8, 28]. Most *Rps9*‐ and *Rps11*‐KD embryos that failed to progress to the blastocyst stage suffered severe fragmentation, whereas *Rpl4‐*, *Rpl13a*‐, and *Rpl19*‐KD embryos exhibited an increase in an arrested morula phenotype as well as severe fragmentation at the blastocyst stage (Figure 2D and Table 1). In contrast, 71.6% of *Rpl39‐*KD embryos developed normally into blastocysts; the morphology of *Rpl39‐*KD embryos that failed to develop into blastocysts was similar to that of control embryos (Figure 2B–D). These results demonstrate that the five RP genes, *Rpl4*, *Rps9*, *Rps11*, *Rpl13a*, and *Rpl19*, are critical for early embryonic development, particularly morula‐to‐blastocyst transition, but *Rpl39* is not. Phenotypic differences among RP gene‐KD embryos, including between *Rpl39*‐KD and the other RP gene‐KD embryos, and between two groups (e.g., *Rps9*/*Rps11*‐KD vs. *Rpl4*/*Rpl13a*/*Rpl19*‐KD) indicate heterogeneity among RPs. Collectively, our results suggest that most RPs play important roles in preimplantation embryonic development, particularly in blastocyst formation. Nevertheless, not all RPs play the same role, and some may have nonessential or heterogeneous functions during early embryonic development.

**TABLE 1:** Knockdown analysis of *Rpl4, Rps9, Rps11, Rpl13a, Rpl19*, and *Rpl39*.

Group	Embryos (total)	Percentage of embryos developed to				Percentage of KD phenotype			
		2‐cell (% ± SEM)	4‐cell (% ± SEM)	Morula (% ± SEM)	Blastocyst (% ± SEM)	Normal blastocyst (% ± SEM)	Abnormal blastocyst (% ± SEM)	Arrested morula (% ± SEM)	Fragmentation (% ± SEM)
Control	193	100 ± 0	98.7 ± 2.5	95.6 ± 6.0	78.5 ± 9.8	78.5 ± 9.8	12.6 ± 7.4	5.3 ± 6.0	3.6 ± 7.0
*Rpl4* KD	42	97.6 ± 3.4	97.6 ± 3.4	88.1 ± 9.9	0 ± 0***	0 ± 0***	2.4 ± 3.4*	26.2 ± 18.7**	71.4 ± 20.2***
*Rps9* KD	38	97.2 ± 3.9	94.8 ± 3.7	86.5 ± 8.2	0 ± 0***	0 ± 0***	4.8 ± 6.7	5.6 ± 7.9	89.7 ± 7.4***
*Rps11* KD	48	100 ± 0	100 ± 0	93.6 ± 0.9	0 ± 0***	0 ± 0***	0 ± 0***	14.1 ± 6.8	85.9 ± 6.8***
*Rpl13a* KD	39	95.2 ± 3.4	95.2 ± 3.4	87.7 ± 5.5	0 ± 0***	0 ± 0***	2.6 ± 3.6*	36.4 ± 8.0***	61.0 ± 11.2***
*Rpl19* KD	36	100 ± 0	100 ± 0	86.8 ± 9.7	0 ± 0***	0 ± 0***	5.6 ± 4.0	30.9 ± 5.7***	63.5 ± 6.4***
*Rpl39* KD	32	100 ± 0	100 ± 0	96.3 ± 5.2	71.6 ± 8.2	71.6 ± 8.2	9.6 ± 1.4	18.8 ± 8.1	0 ± 0

*Note:* Data are presented as means ± SEM. Two‐tailed Student's *t* tests were used for statistical analysis (***p < 0.001, **p < 0.01, *p < 0.05).

### 3.3 Protein Synthesis in RP Gene‐KD Embryos

**FIGURE 3:**
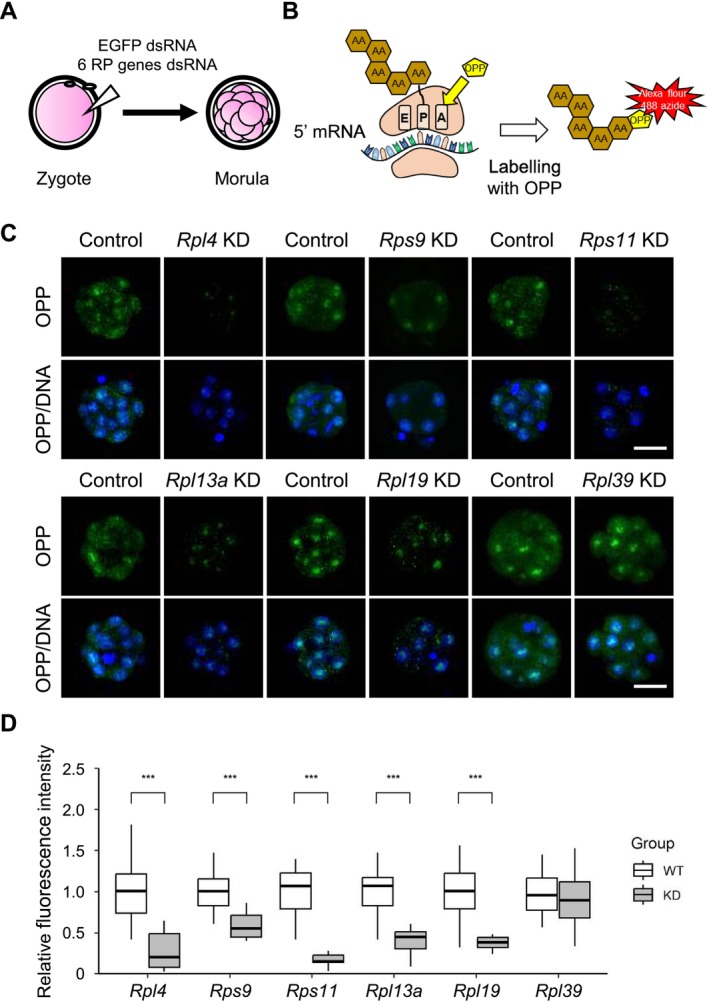
Quantification of *de novo* protein synthesis at the morula stage. (A) Zygotes (20 h post‐hCG injection) were injected with 1 mg/mL of EGFP dsRNA or RP dsRNA. Injected embryos were allowed to develop to the morula stage. (B) Schematic of the OPP assay principle. (C) OPP signals in control and RP gene‐KD embryos, detected 90 h post‐hCG injection. Scale bar, 30 μm. (D) Summary data showing relative *de novo* protein synthesis in control embryos (*n* = 14–22) and KD embryos for *Rpl4* (*n* = 15), *Rps9* (*n* = 14), *Rps11* (*n* = 15), *Rpl13a* (*n* = 15), *Rpl19* (*n* = 14), and *Rpl39* (*n* = 16) used for OPP assays. Data are shown as boxplots in which boxes and whiskers indicate quartiles and the band inside the box denotes the second quartile (median). Values are means ± SD; ***p < 0.001 (three biological replicates, Student's *t* test).

We next investigated whether the six selected RPs regulate *de novo* protein synthesis through ribosome assembly in preimplantation embryos. To measure global protein synthesis in RP gene‐KD embryos, we performed OPP assays at four‐cell, eight‐cell, and morula stages (Figure 3A,B). OPP fluorescence signals were detected in both the nucleus and cytoplasm (Figures 3C, S3 and S4) [31]. Among RP gene‐KD embryos, only *Rpl4*‐KD embryos exhibited a decrease in the amount of newly synthesized protein at the four‐cell stage, and this decrease was relatively modest (~27% less than controls). At the eight‐cell stage, *de novo* protein synthesis was unaffected in any of the RP gene‐KD embryos (Figures S3 and S4). This suggests that none of the six RPs are involved in global *de novo* protein synthesis through the eight‐cell stage. In general, maternal RNAs and proteins are gradually degraded from the two‐cell to the morula stage. Global proteins are likely to be synthesized normally by ribosomes containing these maternal RPs, which have not yet been degraded. In contrast, at the morula stage, overall *de novo* protein synthesis was significantly reduced in KD embryos for five RP genes (*Rpl4*, *Rps9*, *Rps11*, *Rpl13a*, and *Rpl19*), but not in *Rpl39*‐KD embryos (Figure 3C,D). Thus, this strongly suggests that these five RPs are crucial components of ribosomes and participate in ribosome biogenesis, thereby regulating early embryonic development. It is noteworthy that KD embryos showed different degrees of reduction in OPP signal among RP genes (47 ~ 84%) (Figure 3C,D). In contrast, *Rpl39‐KD* embryos showed no changes in global *de novo* protein synthesis, even at the morula stage. This suggests that, unlike other RPs, RPL39 may make a negligible contribution to ribosome biogenesis and thus does not affect early embryonic development, consistent with the relatively normal development of *Rpl39*‐KD embryos (Figure 2). Alternatively, the absence of *Rpl39* could be compensated by other RPs.

### 3.4 Cell Proliferation in RP Gene‐KD Embryos

**FIGURE 4:**
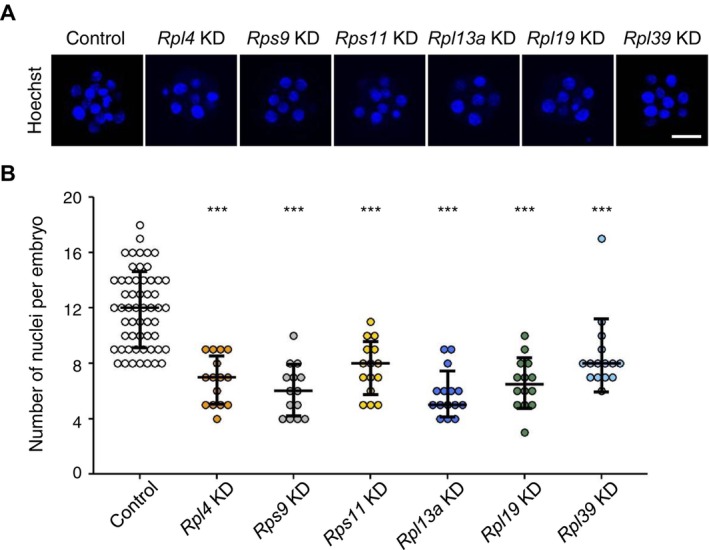
Number of nuclei in six RP gene‐KD embryos. Embryos were injected with EGFP or dsRNA for each of the six RP genes at the zygote stage (20 h post‐hCG injection). (A) Confocal images (Z‐series projections) of embryos stained with Hoechst at 90 post‐hCG injection, used for counting nuclei. Scale bar, 30 μm. (B) Scatter plots of the number of nuclei in control embryos (*n* = 56) and KD embryos for *Rpl4* (*n* = 15), *Rps9* (*n* = 14), *Rps11* (*n* = 15), *Rpl13a* (*n* = 15), *Rpl19* (*n* = 14), and *Rpl39* (*n* = 15). The mean number in each group is indicated by a line. Values are means ± SD; ***p < 0.001 (three biological replicates, Student's *t* test).

**FIGURE 5:**
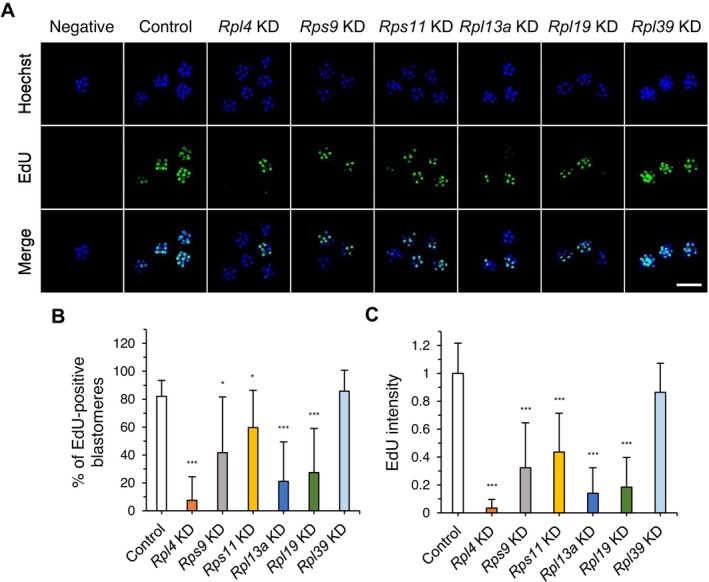
EdU staining analysis of six RP gene‐KD embryos. Embryos were injected with EGFP or dsRNA for each of the six RP genes at the zygote stage (20 h post‐hCG injection). (A) Embryos stained with EdU and Hoechst at 90 h post‐hCG injection. EGFP dsRNA‐injected embryos (*n* = 20) and KD embryos for *Rpl4* (*n* = 12), *Rps9* (*n* = 7), *Rps11* (*n* = 11), *Rpl13a* (*n* = 12), *Rpl19* (*n* = 11), and *Rpl39* (*n* = 9) were used for EdU staining analysis. Scale bar, 100 μm. (B) Percentage of EdU‐positive blastomeres. EdU‐positive blastomeres were counted in Z‐series projections of confocal images. Data are presented as means ± SD; ***p < 0.001, *p < 0.05 (three biological replicates, Student's *t* test). (C) EdU staining intensity, quantified using image J. Data are presented as means ± SD; ***p < 0.001 (three biological replicates, Student's *t* test).

To determine whether—and if so, which—cellular activities were impaired in embryos in which expression of each of the six RP genes was suppressed by KD, we first analyzed cell division and proliferation by counting nuclei in embryos at the morula stage. All RP gene‐KD embryos had fewer nuclei compared with controls (Figure 4). We then examined whether each RP gene‐KD was associated with apoptosis or defects in cell‐cycle progression or cell proliferation. No RP gene‐KDs showed changes in TUNEL signaling during the morula stage, suggesting the absence of apoptosis (Figure S5). Cell proliferation analyses based on EdU staining showed suppressive effects in KD embryos for the same five RP genes (*Rpl4*, *Rps9*, *Rps11*, *Rpl13a*, and *Rpl19*) (Figure 5). *Rpl4*‐, *Rpl13a*‐, and *Rpl19*‐KD embryos displayed a marked reduction in EdU signals, whereas *Rps9*‐ and *Rps11*‐KD embryos showed only slightly reduced EdU signals. Interestingly, the varying degrees of EdU signal among RP gene‐KD embryos correspond to the severity of the KD phenotypes, such as developmental arrest and fragmentation (Figure 2D). In *Rpl39*‐KD embryos, which showed reduced numbers of nuclei (Figure 4), the degree of EdU staining was unchanged (Figure 5). The fact that *Rpl39*‐KD blastocyst‐stage embryos were slightly smaller than control blastocysts, despite their normal appearance, suggests the possibility of a cell proliferation defect with unknown mechanisms (Figure S6). Overall, our data indicate that each RP is involved in cell proliferation to different degrees, revealing a novel aspect of ribosome heterogeneity.

### 3.5 Relationship Between *Rpl39* and *Rpl39l* in Preimplantation Embryos

**FIGURE 6:**
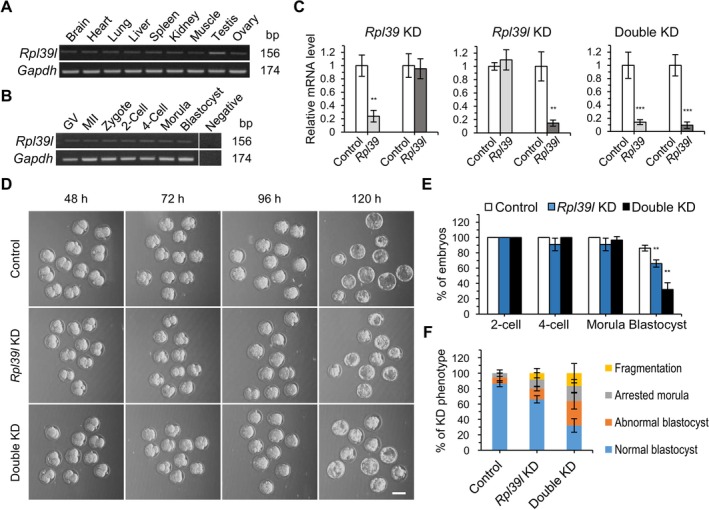
Relationship between *Rpl39* and *Rpl39l* in preimplantation embryos. *Rpl39* and *Rpl39l* expression in (A) various tissues and (B) preimplantation embryos, including oocytes. mRNA expression was determined by qRT‐PCR using cDNA; *Gapdh* was used as a loading control. (C) Relative mRNA levels of *Rpl39* and *Rpl39l* in single‐KD and double‐KD embryos, determined by qRT‐PCR using cDNA synthesized from morula stage embryos at 90 h post‐hCG injection. Values represent means ± SEM; ***p < 0.001, **p < 0.01 (experiments were repeated three times, Student's *t* test). Expression levels of *Rpl39* and *Rpl39l* were normalized to that of *Gapdh*. (D) Micrographs showing each stage in the development of control embryos, *Rpl39l* single‐KD embryos, and *Rpl39l* + *Rpl39* double‐KD embryos at 48, 72, 96, and 120 h post‐hCG injection. Scale bar, 100 μm. (E) Development of embryos, monitored from the two‐cell to the blastocyst stage (up to 120 h post‐hCG injection). EGFP dsRNA‐injected embryos (*n* = 36) and *Rpl39l* single‐KD (*n* = 36) and *Rpl39l* + *Rpl39l* double‐KD (*n* = 31) embryos were used for the analysis. Data are presented as means ± SEM; **p < 0.01 (three biological replicates, Student's *t* test). (F) Control and RP gene‐KD embryos were separated based on morphology at the blastocyst stage. Error bars represent SEM. GV, germinal vesicle oocyte; MII, metaphase II egg; Negative, no template.

Consistent with the above suggestion that another RP could compensate for the KD of *Rpl39*, a paralogous gene, *Rpl39l*, encoding a protein with a sequence that differs from that of RPL39 by only three amino acids (out of a total of 51 amino acids) is known to exist in the mammalian genome. In fact, several studies have shown a compensatory relationship between *Rpl39*/RPL39 and *Rpl39l*/RPL39L in non‐embryonic cells [32, 33]. Therefore, we further investigated the relationship between *Rpl39* and *Rpl39l* in mouse preimplantation embryos. We found that *Rpl39l* was expressed in various tissues, exhibiting the highest expression in the testis, with wide expression in oocytes and preimplantation embryos (Figure 6A,B). We next performed single‐KD and double‐KD analyses of *Rpl39* and *Rpl39l*. Suppression of *Rpl39* expression did not change the expression of *Rpl39l*, or vice versa, at the morula stage (Figure 6C), suggesting the absence of regulatory mechanisms at the mRNA level that serve to compensate for repression of the paralogous genes at the morula stage. However, these RPs may play complementary roles at protein and functional levels. An examination of KD phenotypes in *Rpl39l* single‐KD and *Rpl39l* + *Rpl39* double‐KD embryos showed that both single‐ and double‐KD embryos developed normally to the morula stage. However, approximately 34% of *Rpl39l*‐KD embryos did not develop into blastocysts (Figure 6D,E), a finding that contrasts with the normal blastocyst formation in *Rpl39* single‐KD embryos (see Figure 2). Notably, about 77% of *Rpl39l* + *Rpl39* double‐KD embryos failed to develop to the blastocyst stage (Figure 6D,E). Thus, double KD resulted in a more severe phenotype than either *Rpl39* or *Rpl39l* single KD. These results suggest that both RPL39 and RPL39L are important for early embryonic development and have the potential to compensate for each other. Compared with KD embryos for the other five RPs, *Rpl39l* + *Rpl39* double‐KD embryos showed higher rates of blastocyst formation and lower rates of fragmentation (Figures 2 and 6D,F and Table 2), implying differences in their ribosomal activity.

**TABLE 2:** Knockdown analysis of *Rpl39l* and *Rpl39 + Rpl39l*.

Group	Embryos (total)	Percentage of embryos developed to				Percentage of KD phenotype			
		2‐cell (% ± SEM)	4‐cell (% ± SEM)	Morula (% ± SEM)	Blastocyst (% ± SEM)	Normal blastocyst (% ± SEM)	Abnormal blastocyst (% ± SEM)	Arrested morula (% ± SEM)	Fragmentation (% ± SEM)
Control	36	100 ± 0	100 ± 0	100 ± 0	86.4 ± 4.0	86.4 ± 4.0	7.8 ± 5.9	5.8 ± 4.1	0 ± 0
*Rpl39l* KD	36	100 ± 0	91.0 ± 8.3	91.0 ± 8.3	66.0 ± 4.7**	66.0 ± 4.7**	13.7 ± 2.8	12.22 ± 8.7	8.1 ± 6.0
Double KD	31	100 ± 0	100 ± 0	96.7 ± 4.7	32.1 ± 8.7**	32.1 ± 8.7**	31.8 ± 10.5*	19.4 ± 8.2	16.7 ± 12.5

*Note:* Data are presented as means ± SEM. Two‐tailed Student's *t* tests were used for statistical analysis (**p < 0.01, *p < 0.05).

### 3.6 Ribosomal Function of *Rpl39* and *Rpl39l* in Preimplantation Embryos

**FIGURE 7:**
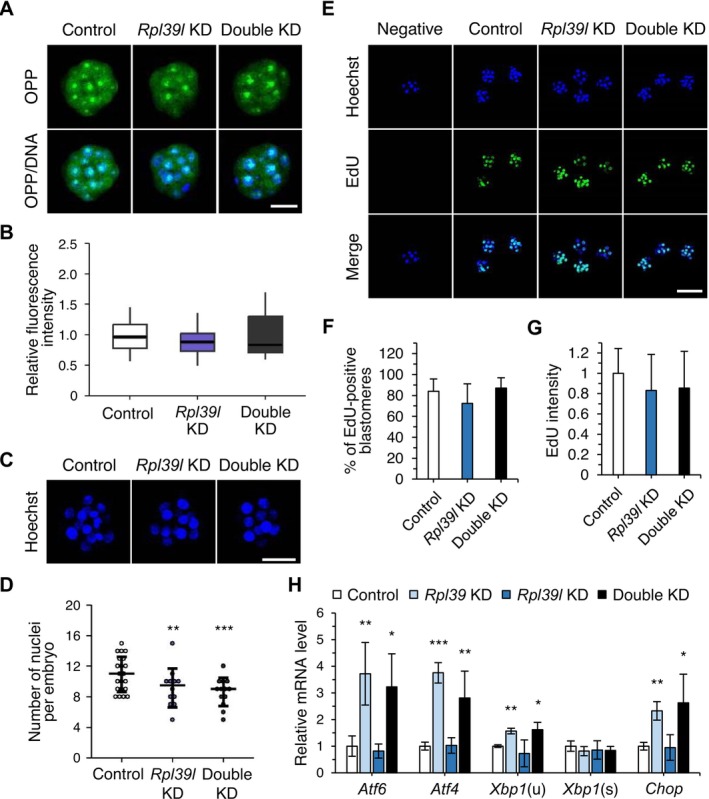
Functions of *Rpl39* and *Rpl39l* in preimplantation embryos. (A) OPP signals in control and RP gene‐KD embryos, detected at 90 h post‐hCG injection. Scale bar, 30 μm. (B) Summary data showing relative *de novo* protein synthesis in control embryos (*n* = 22) and *Rpl39l* single‐KD (*n* = 13) and double‐KD (*n* = 13) embryos used for OPP assays. Data are shown as boxplots, in which boxes and whiskers indicate quartiles and the band inside the box denotes the second quartile (median). Values are means ± SD of three biological replicates. (C) Confocal images (Z‐series projections) of embryos stained with Hoechst at 90 post‐hCG injection used for counting nuclei. Scale bar, 30 μm. (D) Scatter plot of the number of nuclei in control embryos (*n* = 21) and *Rpl39l* single‐KD (*n* = 12) and double‐KD (*n* = 13) embryos. The mean number in each group is indicated by a line. Values are means ± SD; ***p < 0.001, **p < 0.01 (three biological replicates, Student's *t* test). (E) EGFP dsRNA‐injected embryos (*n* = 17) and *Rpl39l* single‐KD (*n* = 12) and double‐KD (*n* = 8) embryos used for EdU staining analysis. Embryos were stained with EdU and Hoechst at 90 h post‐hCG injection. Scale bar, 100 μm. (F) Percentage of EdU‐positive blastomeres. EdU‐positive blastomeres were counted in Z‐series projections of confocal images. Data are presented as means ± SD of three biological replicates. (G) EdU intensity, quantified using image J. Data are presented as means ± SD of three biological replicates. (H) Expression levels of UPR‐related genes, normalized to that of *Gapdh*. cDNA was synthesized from early blastocyst stage embryos at 96 h post‐hCG injection. Values represent means ± SEM; ***p < 0.001, **p < 0.01, *p < 0.5 (three biological replicates, Student's *t* test). *Xbp1*(u), universal *Xbp1*; *Xbp1*(s), spliced *Xbp1*; *Xbp1*, X‐box binding protein 1.

To investigate whether RPL39L is also involved in protein synthesis during early embryonic development, we performed OPP assays at the morula stage. In *Rpl39l* single‐KD and *Rpl39l* + *Rpl39* double‐KD embryos, global *de novo* protein synthesis was not reduced, as was the case in *Rpl39* single‐KD embryos (Figures 3 and 7A,B). These data indicate that neither RPL39 nor RPL39L are involved in ribosome biogenesis. To determine how *Rpl39* and *Rpl39l* KD affected early embryonic development, we counted nuclei and analyzed cell apoptosis and proliferation. Both *Rpl39l*‐KD embryos and double‐KD embryos showed a slight decrease in cell number (Figure 7C,D), but no changes in cell proliferation (EdU staining) or apoptosis (Figures 7E–G and S5). These observations are consistent with the *Rpl39*‐KD phenotype and imply a cell proliferation defect with unknown mechanisms (Figures 4, 5 and S5). *Rpl39l*‐ and double‐KD blastocysts did not differ in size (Figure S6), possibly reflecting the fact that, unlike the case with *Rpl39* KD, *Rpl39l* KD and double KD increased the number of abnormal embryos with several phenotypes rather than reduced‐size blastocysts. These results again suggest that *Rpl39* and *Rpl39l* regulate early embryogenesis through functions distinct from those of other RPs, highlighting the heterogeneity involving the two paralogs.

A recent study reported that either RPL39 or RPL39L is associated with the ribosome exit tunnel in spermatogenic cells, and that protein folding is changed depending on which is incorporated [34]. To determine whether protein folding is altered by *Rpl39* and/or *Rpl39l* KD, we examined changes in the expression of unfolded protein response (UPR) pathway genes in blastocysts. We found that activating transcription factor 6 (*Atf6*), activating transcription factor 4 (*Atf4*), and C/EBP homologous protein (*Chop*) expression were increased in *Rpl39*‐KD embryos and double‐KD embryos (Figure 7H). Although upregulation of UPR genes is not limited to protein misfolding and may also result from increased integrated stress response (ISR), these results strongly suggest that, among UPR pathways, *Rpl39* KD activates protein kinase R‐like ER kinase (PERK) and ATF6 pathways in embryos. Notably, expression of UPR genes was increased only in KD embryos in which *Rpl39* expression was suppressed (*Rpl39*‐KD and *Rpl39l* + *Rpl39*‐KD embryos), but not in *Rpl39l*‐KD embryos. This suggests the existence of a separate group of embryonic proteins whose folding is normally accomplished only by ribosomes containing RPL39. In this case, proteins other than those in this separate group can be folded correctly by either the RPL39‐ribosome or the RPL39L‐ribosome. Taken together, our results suggest that RPL39 and RPL39L regulate early embryonic development through both common and differential control of protein folding during morula‐to‐blastocyst development.

## 4 Discussion

In early embryonic development, *de novo* protein synthesis is crucial for the development from zygote to blastocyst. This *de novo* protein synthesis takes place in ribosomes, and the proteins translated by ribosomes vary depending on the RPs that make up the ribosome. This indicates ribosome heterogeneity, which reflects characteristics such as RP stoichiometry, composition, and the existence of paralogs [17]. In addition, it has recently been discovered that RPs not only assemble ribosomes; they also perform a variety of extra‐ribosomal functions [35]. Despite the diverse functions of RPs, few studies have investigated RPs in mouse preimplantation embryos, and among recent studies that have, only phenotypes associated with some RPs have been investigated. How these RPs regulate early embryonic development has not been examined [9, 26], highlighting the necessity of studying ribosome heterogeneity and extra‐ribosomal functions in mouse implantation embryos.

In this study, we selected six RPs as representatives of the ~82 known RPs and analyzed their functions in early mouse embryonic development by knocking down the corresponding genes. Of the resulting six RP gene‐KD embryos—*Rpl4‐*, *Rps9‐*, *Rps11‐*, *Rpl13a‐*, *Rpl19‐*, and *Rpl39‐*KD—all but *Rpl39‐*KD embryos shared defects in the transition from morula to blastocyst. In this regard, it is not known how much maternal RPs remain during the morula‐to‐blastocyst transition and to what extent they are involved in the transition, although the amounts are relatively small and unaffected by KD. Further studies using RP gene‐knockout (KO) mice are needed to determine the exact function of each RP gene. Despite the phenotypic similarities, there were some differences among RP gene‐KD embryos, and these differences appeared to depend on whether the RP is an LSU or SSU protein. Whereas *Rpl4‐*, *Rpl13a‐*, and *Rpl19*‐KD embryos exhibited significantly inhibited cell proliferation together with fragmentation and arrested morula phenotypes, *Rps9*‐ and *Rps11‐*KD embryos exhibited primarily fragmentation with a relatively weak cell‐proliferation phenotype. Currently, we do not know whether the difference is due to ribosomal subunit‐specific ribosome depletion or to other factors, such as RP‐specific ribosomal functions or extra‐ribosomal functions. The decrease in cell proliferation may result from a decrease in overall protein synthesis, but RPs may also directly regulate cell‐cycle–related proteins. In fact, several studies have shown that cell‐cycle–related proteins are regulated by the extra‐ribosomal functions of RPs [20, 21]. One of the RPs known to be involved in the cell cycle is RPS9 [36]. In addition, RPL4 has been reported to play a role in immune signaling and tumorigenesis [37, 38]; RPS11, RPL19, and RPL39 are involved in tumorigenesis [39, 40, 41]; and RPL13A has been found to regulate inflammation [42, 43]. In particular, studies in KO mouse embryos have demonstrated that loss of RPL13A function blocks blastocyst formation, but the exact mechanism has not been elucidated [44]. These extra‐ribosomal functions may be related to differences in the KD phenotype we found. Thus, just as ribosome heterogeneity in mouse preimplantation embryos requires further study, so do the extra‐ribosomal functions of each RP.

Our analysis of *de novo* protein synthesis found that the extent to which protein expression was reduced varied among RPs. These differences in *de novo* protein synthesis may result from differences in the efficiency of ribosome biogenesis. For example, one previous study found that, depending on which RP was deleted, 20S rRNA might not be produced, it might be produced but not converted to 18S rRNA, or it is produced and converted to 18S rRNA, leading to the production of normal ribosomes [14]. Alternatively, differences may arise indirectly owing to differences in specific proteins regulated by ribosome heterogeneity (i.e., ribosomes composed of different specific RPs) [18, 19]. Follow‐up studies evaluating rRNA maturation in RP gene‐KO embryos are needed, which will allow us to determine the relationship between the RPs and rRNA processing and furthermore whether the RPs regulate subunit‐dependent maturation of rRNAs.

While *Rpl39* KD did not affect early embryonic development, KD of its isoform, *Rpl39l*, slightly reduced the rate of blastocyst development, and *Rpl39l* + *Rpl39* double‐KD resulted in failure of the majority of embryos to develop into normal blastocysts. Unlike the case for the other RPs examined, *Rpl39* and *Rpl39l* KD did not significantly affect *de novo* protein synthesis or cell proliferation. In terms of KD phenotypes, whereas other RP gene‐KD embryos mainly failed to develop into blastocysts, some *Rpl39l* + *Rpl39*‐KD embryos developed into blastocysts, but these blastocysts were abnormal and incomplete. These findings suggest that *Rpl39* and *Rpl39l* have functions different from those of other RPs.

A recent study in spermatogenic cells reported the important finding that RPL39 and RPL39L are associated with the exit tunnel of ribosomes and thus regulate protein folding. Notably, unlike RPL39, RPL39L regulates the folding of male germ cell‐specific proteins, which are known to be rich in cysteine [34]. Another study in mouse embryonic stem cells reported that the dwell time of leucine codons was increased, whereas that of the CAC codon of histidine was decreased in the nascent peptide exit tunnel of *Rpl39l*‐KO ribosomes. It also found that expression of ectoderm markers was increased and expression of endoderm markers was decreased in KO cells [45]. In our study, abnormal blastocyst development was observed relatively weakly in *Rpl39l*‐KD embryos and strongly in *Rpl39l* + *Rpl39* double‐KD embryos. We also found that the UPR pathway was activated in *Rpl39*‐KD and double‐KD embryos at a time point in culture that corresponds to the blastocyst stage in control embryos. On the basis of these results, we speculate that RPL39 and RPL39L may be involved in a protein folding process that is important for blastocyst development in mouse preimplantation embryos. Interestingly, abnormal phenotypes were almost absent in *Rpl39*‐KD embryos despite strong activation of the UPR pathway (protein misfolding signal). In contrast, *Rpl39l*‐KD embryos exhibited some degree of abnormal blastocyst development but lacked misfolded protein signals, and *Rpl39* + *Rpl39l* double‐KD embryos showed both strong abnormal embryogenesis and protein misfolding phenotypes. These seemingly contradictory results suggest that RPL39‐containing and RPL39L‐containing ribosomes may fold different groups of proteins and that these groups may have different functions in blastocyst development.

**FIGURE 8:**
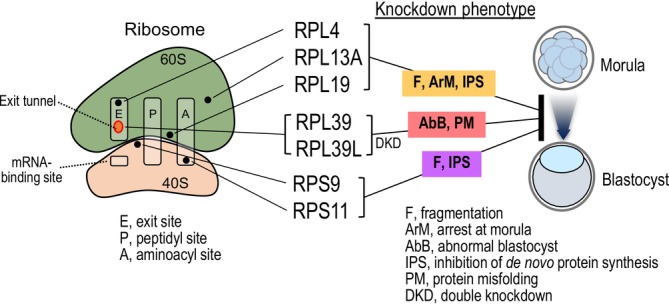
Schematic summary of the current results. KD studies have shown that RPs are important for normal blastocyst formation and development. However, not all proteins contribute equally to embryonic development. This provides a new example of ribosome heterogeneity in preimplantation embryos.

In conclusion, we demonstrate the importance of RPs in mouse preimplantation embryos. We found that different RPs exert differential effects on early embryonic development, revealing ribosome heterogeneity, particularly involving RPL39 and RPL39L (Figure 8). Further studies are needed to identify differences in proteins regulated by RPL39 and RPL39L in early embryonic development. In addition to the RPs investigated in this study, other RPs should also be studied for their functions in mouse early embryonic development. Our study provides the methodological and conceptual foundation for such comprehensive future studies.

## Author Contributions

Chunghee Cho, Seung Jae Lee, and Inchul Choi conceived the study. Seung Jae Lee and Dayeon Kim performed all experiments. Gwidong Han and Seung Pyo Hong contributed reagents or analytic tools. Inchul Choi and Chunghee Cho supervised the work. Seung Jae Lee, Dayeon Kim, and Chunghee Cho wrote the manuscript. All authors have read and agreed to the publication of this manuscript.

## Conflicts of Interest

The authors declare no conflicts of interest.

## Supporting information


**Data S1.** Supporting Information.

Table S2.

## Data Availability

The data that support the findings of this study are available in the Materials and Methodologies, Results, and Supporting Information of this article.

## References

[1] W. Zheng and K. Liu , “Maternal Control of Mouse Preimplantation Development,” in Mouse Development. Results and Problems in Cell Differentiation, vol. 55 (Springer Berlin Heidelberg, 2012), 115–139. 10.1007/978-3-642-30406-4_7 22918804

[2] M. D. White , S. Bissiere , Y. D. Alvarez , and N. Plachta , “Mouse Embryo Compaction,” in Mammalian Preimplantation Development. Current Topics in Developmental Biology, vol. 120 (Elsevier, 2016), 235–258. 10.1016/bs.ctdb.2016.04.005 27475854

[3] M. D. White , J. Zenker , S. Bissiere , and N. Plachta , “Instructions for Assembling the Early Mammalian Embryo,” Developmental Cell 45 (2018): 667–679. 29920273 10.1016/j.devcel.2018.05.013

[4] C. Chazaud and Y. Yamanaka , “Lineage Specification in the Mouse Preimplantation Embryo,” Development 143 (2016): 1063–1074. 27048685 10.1242/dev.128314

[5] J. Nance , “Getting to Know Your Neighbor: Cell Polarization in Early Embryos,” Journal of Cell Biology 206 (2014): 823–832. 25267293 10.1083/jcb.201407064 PMC4178963

[6] Á. Martín and M. J. de los Santos , “The First Choice of the Preimplantation Embryo: How Compaction and Polarity Build Cell Identity,” Medicina Reproductiva y Embriología Clínica 7 (2020): 23–32.

[7] P. Svoboda , “Mammalian Zygotic Genome Activation,” Seminars in Cell & Developmental Biology 84 (2018): 118–126. 29233752 10.1016/j.semcdb.2017.12.006

[8] Y. Gao , X. Liu , B. Tang , et al., “Protein Expression Landscape of Mouse Embryos During Pre‐Implantation Development,” Cell Reports 21 (2017): 3957–3969. 29281840 10.1016/j.celrep.2017.11.111

[9] H. Peng , Y. Zhao , J. Chen , J. Huo , Y. Zhang , and T. Xiao , “Knockdown of Ribosomal Protein S3 Causes Preimplantation Developmental Arrest in Mice,” Theriogenology 129 (2019): 77–81. 30826720 10.1016/j.theriogenology.2019.02.022

[10] Y. Liu , J. Sun , Y. Su , et al., “Nuclear‐Localized Eukaryotic Translation Initiation Factor 1A Is Involved in Mouse Preimplantation Embryo Development,” Journal of Molecular Histology 52 (2021): 965–973. 34405343 10.1007/s10735-021-10014-0

[11] Y. L. Zhang , L. W. Zhao , J. Zhang , et al., “DCAF13 Promotes Pluripotency by Negatively Regulating SUV39H1 Stability During Early Embryonic Development,” EMBO Journal 37 (2018): e98981. 30111536 10.15252/embj.201898981 PMC6138440

[12] Y. Li , J. Tang , X. Ji , et al., “Regulation of the Mammalian Maternal‐To‐Embryonic Transition by Eukaryotic Translation Initiation Factor 4E,” Development 148, no. 12 (2021): 190793. 10.1242/dev.190793 PMC8254863 34013332

[13] A. Ben‐Shem , L. Jenner , G. Yusupova , and M. Yusupov , “Crystal Structure of the Eukaryotic Ribosome,” Science 330 (2010): 1203–1209. 21109664 10.1126/science.1194294

[14] S. Ferreira‐Cerca , G. Poll , P. E. Gleizes , H. Tschochner , and P. Milkereit , “Roles of Eukaryotic Ribosomal Proteins in Maturation and Transport of Pre‐18S rRNA and Ribosome Function,” Molecular Cell 20 (2005): 263–275. 16246728 10.1016/j.molcel.2005.09.005

[15] D. Li and J. Wang , “Ribosome Heterogeneity in Stem Cells and Development,” Journal of Cell Biology 219, no. 6 (2020): e202001108. 32330234 10.1083/jcb.202001108 PMC7265316

[16] D. M. Gay , A. H. Lund , and M. D. Jansson , “Translational Control Through Ribosome Heterogeneity and Functional Specialization,” Trends in Biochemical Sciences 47 (2022): 66–81. 34312084 10.1016/j.tibs.2021.07.001

[17] K. Norris , T. Hopes , and J. L. Aspden , “Ribosome Heterogeneity and Specialization in Development,” Wiley Interdisciplinary Reviews: RNA 12, no. 4 (2021): e1644. 33565275 10.1002/wrna.1644 PMC8647923

[18] N. Kondrashov , A. Pusic , C. R. Stumpf , et al., “Ribosome‐Mediated Specificity in Hox mRNA Translation and Vertebrate Tissue Patterning,” Cell 145, no. 3 (2011): 383–397. 21529712 10.1016/j.cell.2011.03.028 PMC4445650

[19] Z. Shi , K. Fujii , K. M. Kovary , et al., “Heterogeneous Ribosomes Preferentially Translate Distinct Subpools of mRNAs Genome‐Wide,” Molecular Cell 67, no. 1 (2017): 71–83. 28625553 10.1016/j.molcel.2017.05.021 PMC5548184

[20] B. Chen , W. Zhang , J. Gao , et al., “Downregulation of Ribosomal Protein S6 Inhibits the Growth of Non‐Small Cell Lung Cancer by Inducing Cell Cycle Arrest, Rather Than Apoptosis,” Cancer Letters 354, no. 2 (2014): 378–389. 25199762 10.1016/j.canlet.2014.08.045

[21] S. Dutt , A. Narla , K. Lin , et al., “Haploinsufficiency for Ribosomal Protein Genes Causes Selective Activation of p53 in Human Erythroid Progenitor Cells,” Blood 117 (2011): 2567–2576. 21068437 10.1182/blood-2010-07-295238 PMC3062351

[22] J. Lv , X. R. Huang , J. Klug , et al., “Ribosomal Protein S19 Is a Novel Therapeutic Agent in Inflammatory Kidney Disease,” Clinical Science 124 (2013): 627–637. 23252627 10.1042/CS20120526

[23] K.‐H. Lim , K.‐H. Kim , S. I. Choi , et al., “RPS3a Over‐Expressed in HBV‐Associated Hepatocellular Carcinoma Enhances the HBx‐Induced NF‐κB Signaling via Its Novel Chaperoning Function,” PLoS One 6, no. 8 (2011): e22258. 21857917 10.1371/journal.pone.0022258 PMC3156704

[24] J. J. Manfredi , “The Mdm2–p53 Relationship Evolves: Mdm2 Swings Both Ways as an Oncogene and a Tumor Suppressor,” Genes & Development 24 (2010): 1580–1589. 20679392 10.1101/gad.1941710 PMC2912554

[25] X. Guo , Y. Shi , Y. Gou , et al., “Human Ribosomal Protein S13 Promotes Gastric Cancer Growth Through Down‐Regulating p27Kip1,” Journal of Cellular and Molecular Medicine 15, no. 2 (2011): 296–306. 19912438 10.1111/j.1582-4934.2009.00969.x PMC3822796

[26] H. Matsson , E. J. Davey , N. Draptchinskaia , et al., “Targeted Disruption of the Ribosomal Protein S19 Gene Is Lethal Prior to Implantation,” Molecular and Cellular Biology 24 (2004): 4032–4037. 15082795 10.1128/MCB.24.9.4032-4037.2004 PMC387766

[27] S.‐J. Lee , J. Kim , G. Han , S.‐P. Hong , D. Kim , and C. Cho , “Impaired Blastocyst Formation in Lnx2‐Knockdown Mouse Embryos,” International Journal of Molecular Sciences 24 (2023): 1385. 36674899 10.3390/ijms24021385 PMC9867088

[28] B. Lv , C. Liu , Y. Chen , et al., “Light‐Induced Injury in Mouse Embryos Revealed by Single‐Cell RNA Sequencing,” Biological Research 52 (2019): 1–8. 31466525 10.1186/s40659-019-0256-1 PMC6716870

[29] G. Yusupova and M. Yusupov , “Crystal Structure of Eukaryotic Ribosome and Its Complexes With Inhibitors,” Philosophical Transactions of the Royal Society of London. Series B, Biological Sciences 372 (2017): 20160184. 28138070 10.1098/rstb.2016.0184 PMC5311928

[30] N. Slavov , S. Semrau , E. Airoldi , B. Budnik , and A. van Oudenaarden , “Differential Stoichiometry Among Core Ribosomal Proteins,” Cell Reports 13 (2015): 865–873. 26565899 10.1016/j.celrep.2015.09.056 PMC4644233

[31] N. Uozumi , H. Matsumoto , and H. Saitoh , “Detection of O‐Propargyl‐Puromycin With SUMO and Ubiquitin by Click Chemistry at PML‐Nuclear Bodies During Abortive Proteasome Activities,” Biochemical and Biophysical Research Communications 474 (2016): 247–251. 27125456 10.1016/j.bbrc.2016.03.155

[32] Q. Zou , L. Yang , R. Shi , Y. Qi , X. Zhang , and H. Qi , “Proteostasis Regulated by Testis‐Specific Ribosomal Protein RPL39L Maintains Mouse Spermatogenesis,” iScience 24 (2021): 103396. 34825148 10.1016/j.isci.2021.103396 PMC8605100

[33] Q. Zou and H. Qi , “Deletion of Ribosomal Paralogs Rpl39 and Rpl39l Compromises Cell Proliferation via Protein Synthesis and Mitochondrial Activity,” International Journal of Biochemistry & Cell Biology 139 (2021): 106070. 34428590 10.1016/j.biocel.2021.106070

[34] H. Li , Y. Huo , X. He , et al., “A Male Germ‐Cell‐Specific Ribosome Controls Male Fertility,” Nature 612 (2022): 725–731. 36517592 10.1038/s41586-022-05508-0

[35] X. Xiong , Y. Zhao , H. He , and Y. Sun , “Ribosomal Protein S27‐Like and S27 Interplay With p53‐MDM2 Axis as a Target, a Substrate and a Regulator,” Oncogene 30 (2011): 1798–1811. 21170087 10.1038/onc.2010.569 PMC3077453

[36] Y. Iizumi , M. Oishi , T. Taniguchi , W. Goi , Y. Sowa , and T. Sakai , “The Flavonoid Apigenin Downregulates CDK1 by Directly Targeting Ribosomal Protein S9,” PLoS One 8 (2013): e73219. 24009741 10.1371/journal.pone.0073219 PMC3756953

[37] L. Green , B. Houck‐Loomis , A. Yueh , and S. P. Goff , “Large Ribosomal Protein 4 Increases Efficiency of Viral Recoding Sequences,” Journal of Virology 86 (2012): 8949–8958. 22718819 10.1128/JVI.01053-12 PMC3416150

[38] A. Egoh , S. Nosuke Kanesashi , C. Kanei‐Ishii , T. Nomura , and S. Ishii , “Ribosomal Protein L4 Positively Regulates Activity of Ac‐Myb Proto‐Oncogene Product,” Genes to Cells 15 (2010): 829–841. 20604807 10.1111/j.1365-2443.2010.01421.x

[39] C. Zhou , J. Sun , Z. Zheng , et al., “High RPS11 Level in Hepatocellular Carcinoma Associates With Poor Prognosis After Curative Resection,” Annals of Translational Medicine 8, no. 7 (2020): 466. 32395510 10.21037/atm.2020.03.92 PMC7210141

[40] K. Kuroda , M. Takenoyama , T. Baba , et al., “Identification of Ribosomal Protein L19 as a Novel Tumor Antigen Recognized by Autologous Cytotoxic T Lymphocytes in Lung Adenocarcinoma,” Cancer Science 101 (2010): 46–53. 19799608 10.1111/j.1349-7006.2009.01351.x PMC11159900

[41] B. Dave , D. D. Gonzalez , Z.‐B. Liu , et al., “Role of RPL39 in Metaplastic Breast Cancer,” Journal of the National Cancer Institute 109 (2017): djw292. 28040796 10.1093/jnci/djw292 PMC6245334

[42] B. Mazumder , P. Sampath , V. Seshadri , R. K. Maitra , P. E. DiCorleto , and P. L. Fox , “Regulated Release of L13a From the 60S Ribosomal Subunit as a Mechanism of Transcript‐Specific Translational Control,” Cell 115 (2003): 187–198. 14567916 10.1016/s0092-8674(03)00773-6 PMC13188775

[43] K. Vyas , S. Chaudhuri , D. W. Leaman , et al., “Genome‐Wide Polysome Profiling Reveals an Inflammation‐Responsive Posttranscriptional Operon in Gamma Interferon‐Activated Monocytes,” Molecular and Cellular Biology 29 (2009): 458–470. 19001086 10.1128/MCB.00824-08 PMC2612521

[44] R. Kour , J. Kim , A. Roy , et al., “Loss of Function of Ribosomal Protein L13a Blocks Blastocyst Formation and Reveals a Potential Nuclear Role in Gene Expression,” FASEB Journal 37 (2023): e23275. 37902531 10.1096/fj.202301475R PMC10999073

[45] A. Banerjee , M. Ataman , M. J. Smialek , et al., “Ribosomal Protein RPL39L Is an Efficiency Factor in the Cotranslational Folding of a Subset of Proteins With Alpha Helical Domains,” Nucleic Acids Research 52 (2024): 9028–9048. 39041433 10.1093/nar/gkae630 PMC11347166

