# Supplementary material for: Functional Characterization and Heterogeneity Analysis of Ribosomal Proteins in Mouse Preimplantation Embryos
Source: FASEB J. 2025 May 27;39(11):e70662. doi: 10.1096/fj.202500574RR (PMC12107510; doi:10.1096/fj.202500574RR)
Supplement: Supplementary file 1 — Data S1. Supporting Information. [file FSB2-39-e70662-s002.pdf]

Figure S1

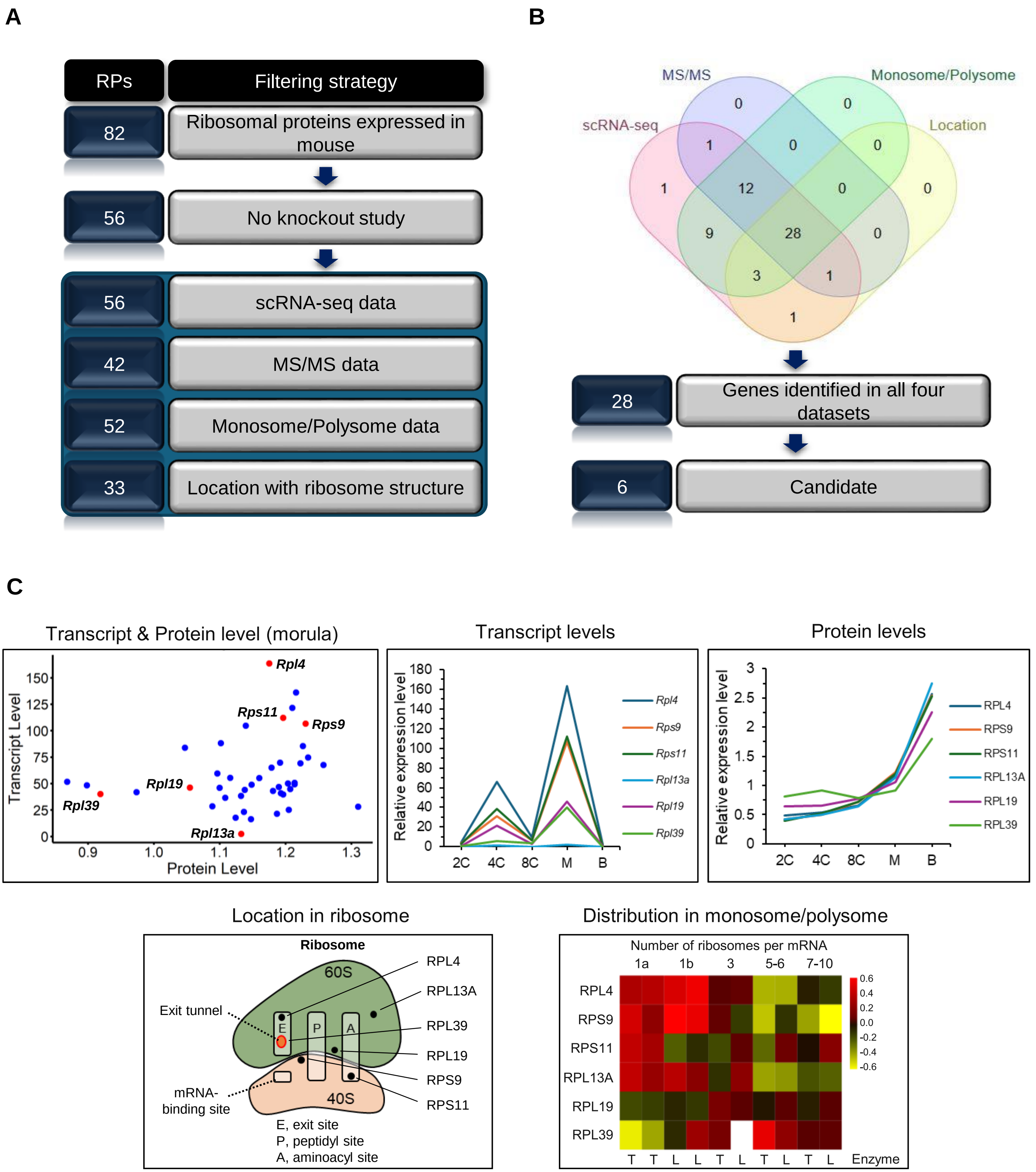

Figure S1. Selection of RPs. (A) RPs analyzed in this study were based on expression levels (transcript and protein), locations within the ribosome structure, and relative levels in monosomes and polysomes among 56 RPs that had not been functionally studied. (B) A total of 28 RPs had these four data information, and six RPs were arbitrarily selected for study. (C) Based on the four criteria above, the six RPs do not lean toward a specific part in each criterion. 2C, 2 cell; 4C, 4 cell; 8C, 8 cell; M, morula; B, blastocyst; T, trypsin; L, lys-C

Figure S2

A

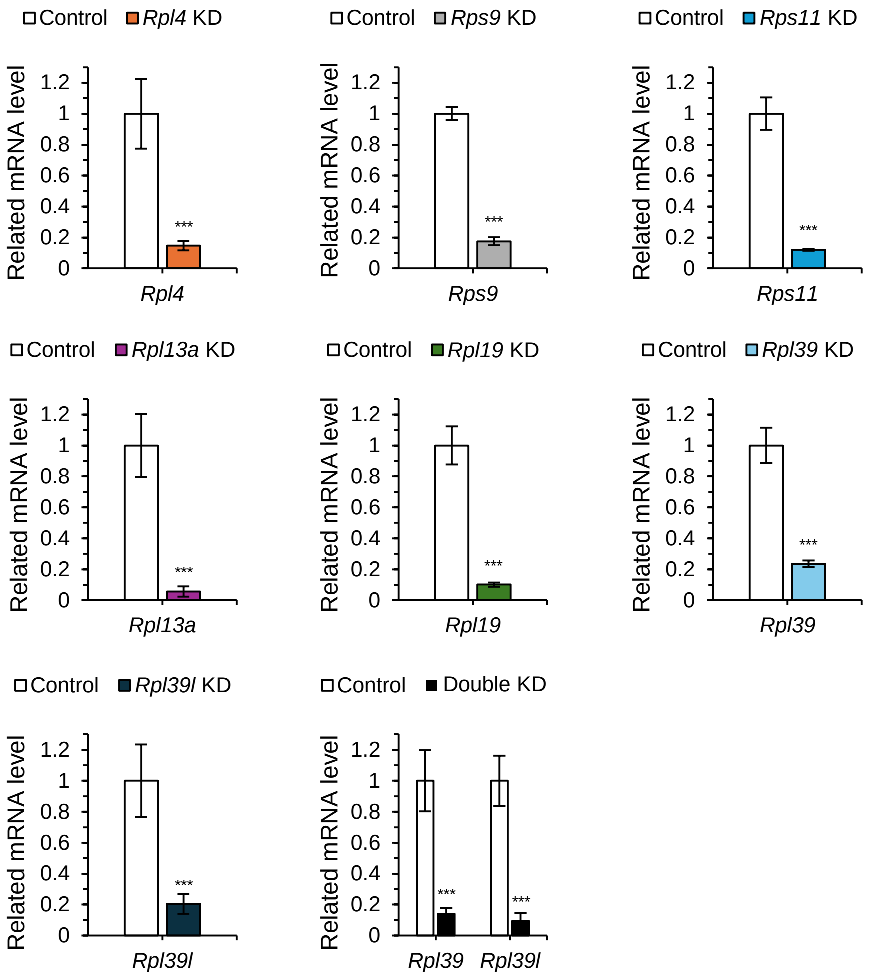

Figure S2. Knockdown effect of dsRNA at the 2-cell stage. Zygotes (20 h post hCG injection) were injected with 1 mg/ml of RP gene dsRNA or 1 mg/ml of EGFP dsRNA (injection control). A) Expression levels of RP genes, normalized to that of *Gapdh*. cDNA was synthesized from 2-cell stage embryos at 48 h post hCG injection. Values represent means ± SEM; $^{***}P < 0.001$ (experiments were repeated three times, Student's *t* test).

Figure S3

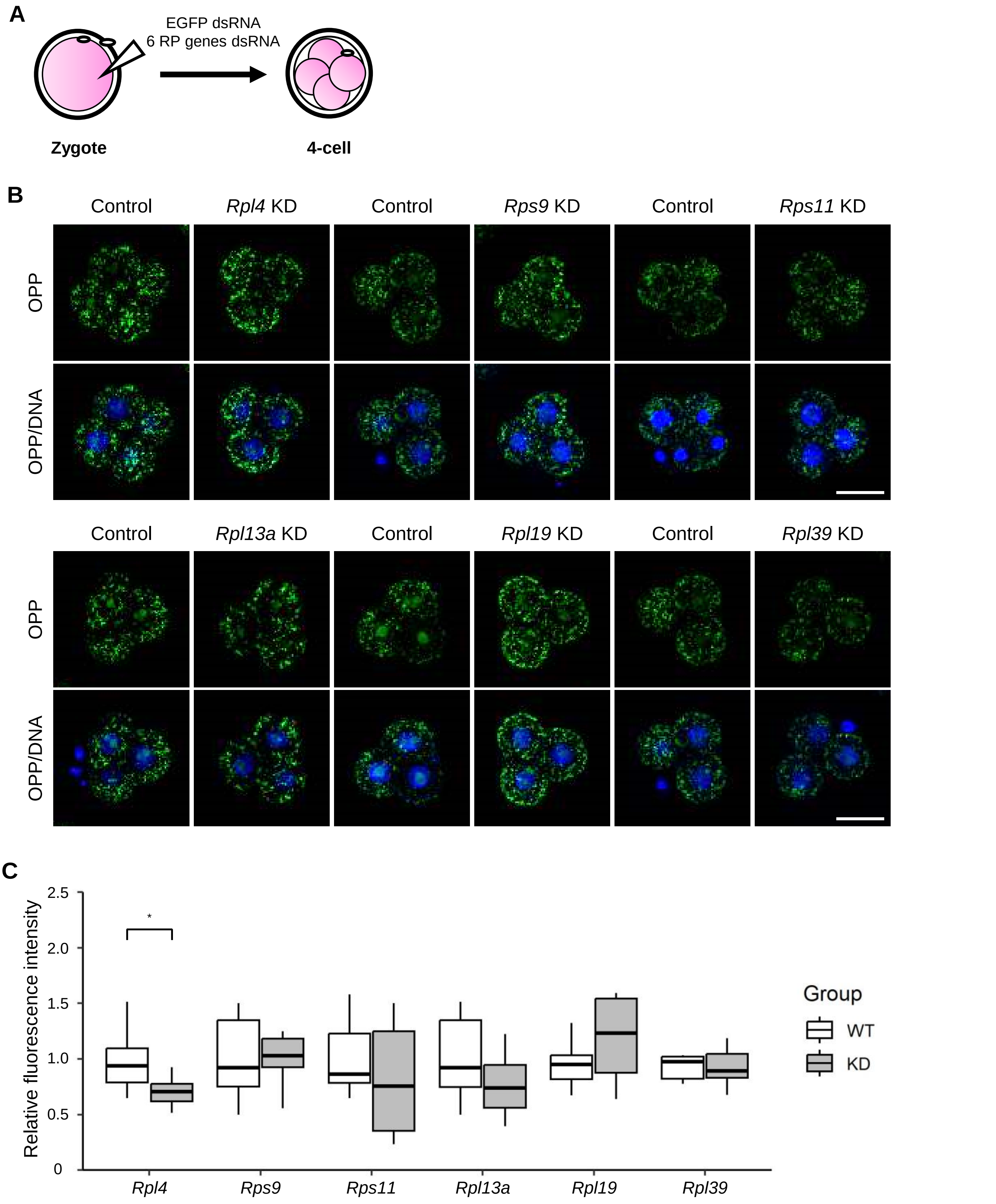

Figure S3. Quantification of de novo protein synthesis in 4-cell stage embryos. A) Overview of injection procedure. Zygotes (20 h post hCG injection) were injected with 1 mg/ml of EGFP dsRNA or RP dsRNA and allowed to develop to the 4-cell stage. B) OPP signals in control and RP gene-KD embryos, detected at 64 h post hCG injection. Scale bar, 30 μm. C) Summary data showing relative de novo protein synthesis in EGFP dsRNA-injected embryos (n=8-12) and KD embryos for *Rpl4* (n=14), *Rps9* (n=10), *Rps11* (n=7), *Rpl13a* (n=10), *Rpl19* (n=9), and *Rpl39* (n=9) used for OPP assays. Data are shown as boxplots in which boxes and whiskers indicate quartiles and the band inside the box denotes the second quartile (median). Values are means ± SD; **P* < 0.05 (three biological replicates, Student's *t* test).

Figure S4

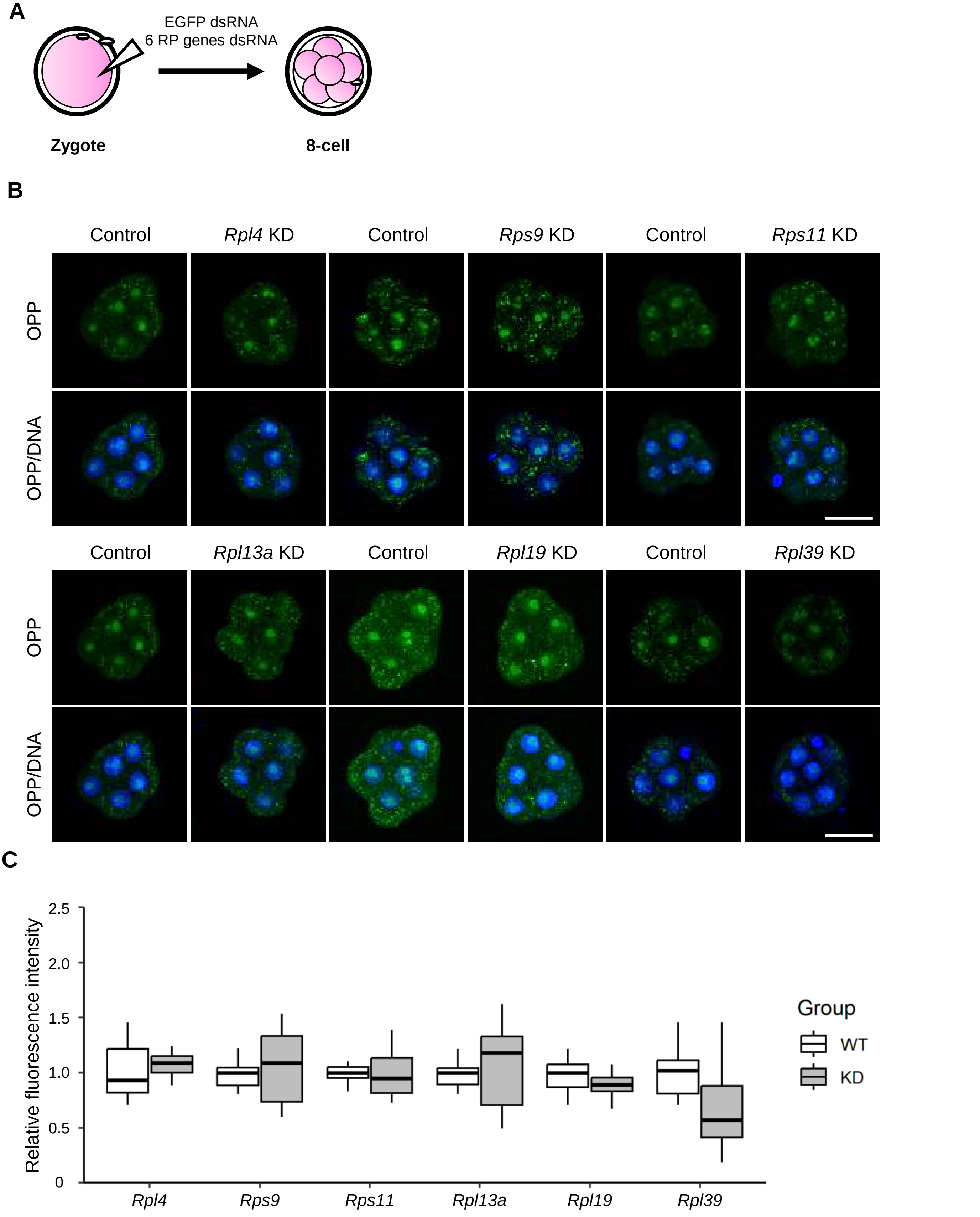

Figure S4. Quantification of de novo protein synthesis in 8-cell stage embryos. Zygotes (20 h post hCG injection) were injected with 1 mg/ml of EGFP dsRNA or RP dsRNA. Injected embryos were allowed to develop to the 8-cell stage. B) OPP signals in control and RP gene-KD embryos, detected at 70 h post hCG injection. Scale bar, 30 μm. C) Summary data showing relative de novo protein synthesis in EGFP dsRNA-injected embryos (n=14-15) and KD embryos of *Rpl4* (n=12), *Rps9* (n=14), *Rps11* (n=11), *Rpl13a* (n=9), *Rpl19* (n=13), and *Rpl39* (n=12) used for OPP assay. Data are shown as boxplots in which boxes and whiskers indicate quartiles and the band inside the box denotes the second quartile (median). Values are means ± SD of three biological replicates.

Figure S5

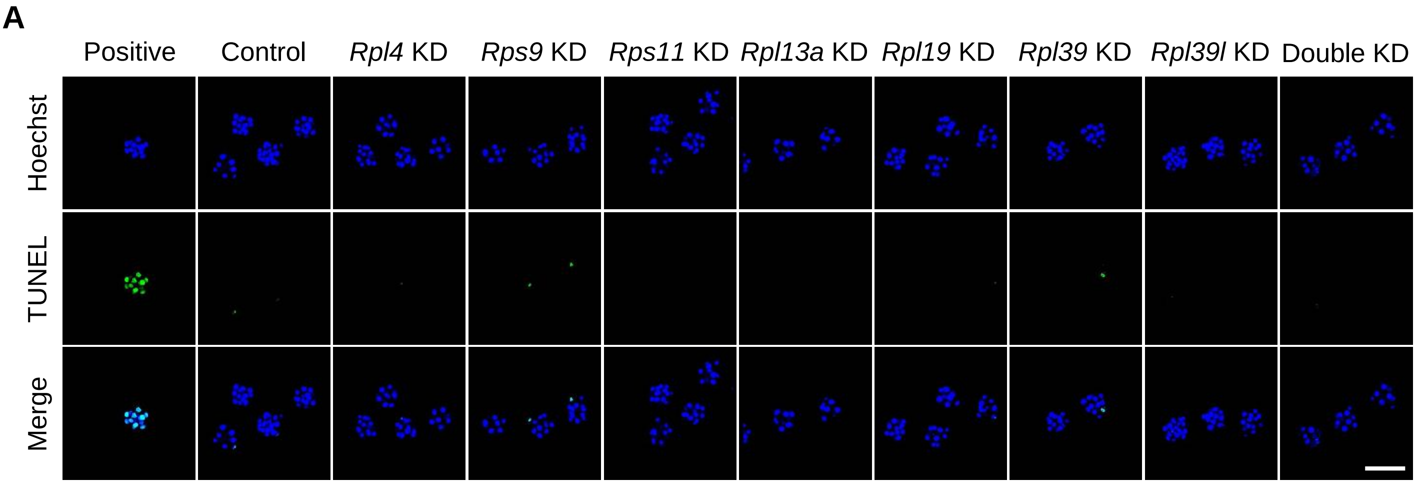

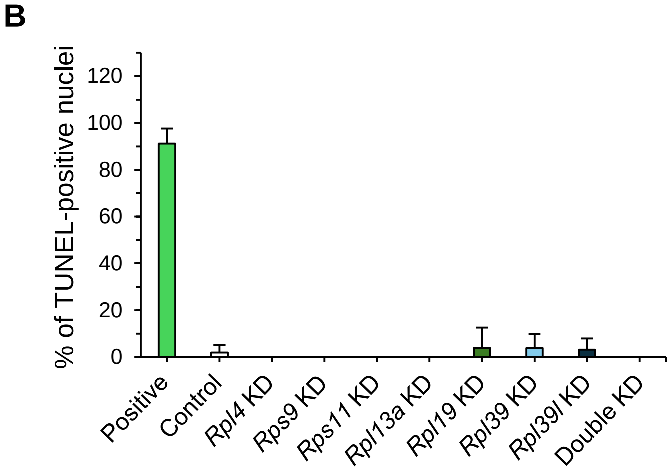

Figure S5. TUNEL staining in eight KD embryos of RP genes (6 RP genes + single- and double-KD of isoform genes). Embryos were injected with EGFP or dsRNA for each of eight RP genes/isoform(s) at the zygote stage (20 h post-hCG injection). A) Embryos stained with TUNEL and Hoechst at 90 h post hCG injection. DNase I-treated embryos (n=6), EGFP dsRNA-injected embryos (n=12), and KD embryos for *Rpl4* (n=12), *Rps9* (n=9), *Rps11* (n=11), *Rpl13a* (n=9), *Rpl19* (n=10), *Rpl39* (n=10), *Rpl39l* (n=12), and *Rpl39l*+*Rpl39* (n=10) were used for TUNEL staining. Scale bar, 100 µm. B) Summary data showing TUNEL-positive blastomeres counted in Z-series projections of confocal images. Data are presented as means ± SD of three biological replicates.

Figure S6

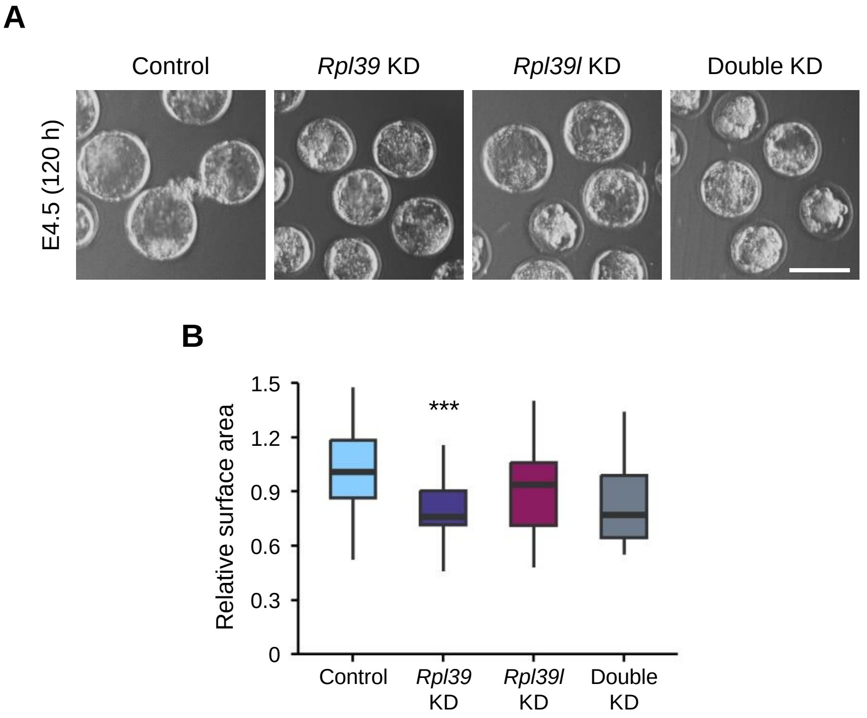

Figure S6. Cell size of *Rpl39-* and *Rpl39l-KD* embryos at the blastocyst stage. Zygotes (20 h post hCG injection) were injected with 1 mg/ml of dsRNA for each RP gene or 1 mg/ml of EGFP dsRNA (injection control). A) Micrographs showing blastocyst stage in the development of control, *Rpl39* and *Rpl39l* single-KD, and *Rpl39l*+*Rpl39l* double-KD embryos, observed at 120 h post hCG injection. Scale bar, 100 µm. EGFP dsRNA-injected embryos (n=58) and *Rpl39* single-KD (n=23), *Rpl39l* single-KD (n=24), and *Rpl39*+*Rpl39l* double-KD (n=10) embryos were used for morphological analyses. B) Relative surface area for injection control and *Rpl39* and *Rpl39l* single- and double-KD embryos. Area was estimated using Image J. Values represent means ± SD; ****P* < 0.001 (experiments were repeated three times, Student's *t* test).

Table S1. List of primers used for RT/qRT-PCR

Gene	Size (bp)	Forward primer	Reverse primer
Gapdh[a,b]	174	5'- TCCGTGTTCCTACCCCCAATG -3'	5'- GGGAGTTGCTGTTGAAGTCGC -3'
Gapdh[a]	986	5'- TGAAGGTCGGTGTGAACGGATTTGGC -3'	5'- CATGTAGGCCATGAGGTCCACCAC -3'
Rpl4[a]	612	5'- TGAGAGCTCTGGCAAGGGCAAAATGAG -3'	5'- GCCGGTACCTCCACCTTCTTTCCTTTC -3'
Rps9[a]	396	5'- TAAGAGCTCAGACCCTTCGAGAAGTCG -3'	5'- ATAGGTACCGGGATGTTCACCACCTGC -3'
Rps11[a]	471	5'- GCCGAATTCTTACCAAAAGCAGCCTAC -3'	5'- GCCGATATCCCTTAGAACTTCTGAAAC -3'
Rpl13a[a]	422	5'- TAGGAGCTCCCAGAAAGTTTGCTTACC -3'	5'- TATGGTACCGCTCAACTGATTCATCCC -3'
Rpl19[a]	358	5'- CTTGAGCTCAGCCTGTGACTGTCCATTCC -3'	5'- GTCGGTACCGCGCTTTCGTGCTTCCTT -3'
Rpl39[a]	326	5'- CTTGAGCTCTCTTCTCCATTCCTCCGCCA -3'	5'- GCTGGTACCGACACGAAGAGTCTCCAGGTTT -3'
Rpl39l[a]	528	5'- TTAGGATCCAAATCCACCCTGCCTTTCC -3'	5'- TGCGAATTCTCTGGACTTGTCTCAACCC -3'
Rpl4[b]	131	5'- GCCCAGAAATCCAAAGAGCCC -3'	5'- ATTCCTGCGCATAGTCTTGGC -3'
Rps9[b]	97	5'- CAACGTCACATTAGGGTCCGC -3'	5'- GAGAACGGAGGGAGAAGTCG -3'
Rps11[b]	151	5'- TCAGACGGAGCGTGCTTACCAAAA -3'	5'- GGTACCCTCAATAGCCTCTTTAGG -3'
Rpl13a[b]	129	5'- AGCCTACCAGAAAGTTTGC -3'	5'- GCTTCTTCTTCCGATAGTGC -3'
Rpl19[b]	126	5'- TGCCAACTCCCGTCAGCAG -3'	5'- ATATGCCTGCCCTTCCGTC -3'
Rpl39[b]	163	5'- CTGGTAACAAAATCAGGTACAACTC -3'	5'- ACACGAAGAGTCTCCAGGTTT -3'
Rpl39l[b]	152	5'- CTTCTCACAAGACCTTCAGG -3'	5'- TTATAGACCCAATTTGGTTCGT -3'
Atf6[b]	190	5'- TCGCCTTTTAGTCCGGTTCTT -3'	5'- GGCTCCATAGGTCTGACTCC -3'
Atf4[b]	119	5'- TTAGAGCTAGGCAGTGAAGTT -3'	5'- CTGTCATTGTCAGAGGGAGT -3'
Xbp1(u)[b]	139	5'- CAGACTATGTGCACCTCTGC -3'	5'- CAGGGTCCAACTTGTCCAGAAT -3'
Xbp1(s)[b]	129	5'- CTGAGTCCGCAGCAGGTG -3'	5'- CAGGGTCCAACTTGTCCAGAAT -3'
Chop[b]	132	5'- GAGTCCCTGCCTTTCACCTT -3'	5'- TTCCTCTTCGTTTCCTGGGG -3'

[a] RT-PCR, [b] qRT-PCR
